# Changes in Treatment of Very Elderly Patients Six Weeks after Discharge from Geriatrics Department

**DOI:** 10.3390/geriatrics5030044

**Published:** 2020-07-29

**Authors:** Mélanie Dipanda, Jérémy Barben, Gilles Nuémi, Lucie Vadot, Valentine Nuss, Jérémie Vovelle, Alain Putot, Patrick Manckoundia

**Affiliations:** 1 “Pôle Personnes Âgées”, Hospital of Champmaillot, Dijon Bourgogne University Hospital, 21079 Dijon, France; melanie.dipanda@chu-dijon.fr (M.D.); jeremy.barben@chu-dijon.fr (J.B.); valentine.nuss@chu-dijon.fr (V.N.); jeremie.vovelle@chu-dijon.fr (J.V.); alain.putot@chu-dijon.fr (A.P.); 2 Department of Biostatistics and Bioinformatics, Dijon Bourgogne University Hospital, 21079 Dijon, France; gilles.nuemi@chu-dijon.fr; 3 Department of Pharmacy, Research and Vigilance, Dijon Bourgogne University Hospital, 21079 Dijon, France; lucie.vadot@chu-dijon.fr; 4 INSERM U-1093, Cognition, Action and Sensorimotor Plasticity, University of Burgundy Franche-Comté, 21079 Dijon, France

**Keywords:** care continuity, elderly, polypharmacy, therapeutic optimization

## Abstract

We assessed the prescriptions of patients hospitalized in a geriatric unit and subsequently discharged. This prospective and observational study was conducted over a two-month period in the geriatrics department (acute and rehabilitation units) of a university hospital. Patients discharged from this department were included over a two-month period. Prescriptions were analyzed at admission and discharge from the geriatrics department (DGD), and six weeks after DGD. We included 209 patients, 63% female, aged 86.8 years. The mean number of medications prescribed was significantly higher at DGD than at admission (7.8 vs. 7.1, *p* = 0.003). During hospitalization, 1217 prescriptions were changed (average 5.8 medications/patient): 52.8% were initiations, 39.3% were discontinuations, and 7.9% were dose adjustments. A total of 156 of the 209 patients initially enrolled completed the study. Among these patients, 81 (51.9%) had the same prescriptions six weeks after DGD. In univariate analysis, medications were changed more frequently in patients with cognitive impairment (*p* = 0.04) and in patients for whom the hospital report did not indicate in-hospital modifications (*p* = 0.007). Multivariate analysis found that six weeks after DGD, there were significantly more drug changes for patients for whom there were changes in prescription during hospitalization (*p* < 0.001). A total of 169 medications were changed (mean number of medications changed per patient: 1.1): 52.7% discontinuations, 34.3% initiations, and 13% dosage modifications. The drug regimens were often changed during hospitalization in the geriatrics department, and a majority of these changes were maintained six weeks after DGD. Improvements in patient adherence and hospital-general practitioner communication are necessary to promote continuity of care and to optimize patient supervision after hospital discharge.

## 1. Introduction

The multimorbidity of people aged 80 years or more [1] complicates the work of health professionals and the organization of care [2]. In addition, because individuals with multiple conditions are generally prescribed more medications, there is an increased risk of drug interactions, nonadherence to treatment, and drug side effects [3,4,5].

Hospitalization is an opportunity for the care team to re-evaluate the prescriptions of individuals in their care, and it has been shown that hospitalization in an acute geriatrics unit (AGU) can reduce the prevalence of inappropriate medication use [6,7]. Once a patient has been hospitalized, the care team will generally analyze the existing prescriptions and modify the drug regimens as appropriate [8].

In order to secure the transition between primary and secondary care and to promote care continuity, medication reconciliation has been developed. It is the process of comparing a patient’s required medication to all of the medication that he/she has been taking, and it has been developed to avoid errors such as omission, duplication, dosing errors and drug interactions [9].

Medication reconciliation is performed by hospital pharmacists when a patient is newly admitted to our AGU. If a patient recently treated in our AGU is readmitted sometime later, we sometimes find significant changes between the medications listed on the discharge records and those observed at readmission.

Only very few studies have focused on changes to drug prescriptions during hospitalization and/or after discharge in a very elderly population (≥85 years) [8,10,11]. In one study, Rouch et al., showed that general practitioners (GPs) maintained a high rate of adherence (83% of continuations) to the treatment modifications suggested at patient discharge from hospital [10]. Another qualitative study attempted to elucidate the reasons behind the poor continuity of medication changes for geriatric patients by GPs [11].

In view of the lack of literature, we conducted a study in order to assess the changes in the prescription of very elderly patients admitted and subsequently discharged from our geriatrics department, and to identify the reasons for these changes. The current challenge is to optimize patient management in this context. Reaching this objective will require, in our opinion, both excellent relationships between hospital physicians, geriatricians, and GPs, as well as improved treatment compliance in very elderly patients.

## 2. Methods

### 2.1. Study Design

A prospective and observational study was conducted over a two-month period, from 1 May to 1 July 2016, in the geriatrics department, (AGU and geriatric rehabilitation unit (GRU)), of our university hospital in Dijon, France.

This study was performed in accordance with the Declaration of Helsinki and National standards. The Ethics Committee of our institution was consulted and approved this study.

### 2.2. Population

#### 2.2.1. Sampling Process

Patients who met the criteria for inclusion were consecutively included by the attending physician of the inpatient unit from 1 May to 1 July 2016. The sample thus formed was followed during hospitalization and six weeks after discharge from the geriatrics department (DGD).

#### 2.2.2. Inclusion Criteria

All elderly patients discharged from the geriatrics department to home were included in the study.

#### 2.2.3. Exclusion Criteria

Patients with the following criteria were not included: transferred from the geriatrics department to other medical, surgery or rehabilitation departments, subjects discharged for home care, and/or end-of-life and/or palliative care, and patients discharged without medical approval.

#### 2.2.4. Exclusion Criteria at Six Weeks

Patients were excluded six weeks after DGD if they had not been in contact with their GP, if they were readmitted to hospital, or in case of death.

### 2.3. Collected Data

Preadmission data were collected as usual from the GP’s letter referring the patients to the hospital, from interviews with patients, their caregivers, and GPs, as well as the digital patient records compiled during previous hospitalizations. Data for the current hospitalization were collected by the physician responsible for patient management. Six weeks after DGD, data were collected through interviews with patients, their caregivers, and GPs. A standardized questionnaire was completed at DGD then over the phone six weeks later.

We analyzed all prescribed medications, including oral nutritional supplements (ONS). Drugs were classified according to the International Anatomical, Therapeutic and Chemical classification of the European Pharmaceutical Market Research Association 5 [12].

At discharge, the following data were collected for each patient: age, gender, residence before and after hospitalization, medical history, cause of hospitalization, where they were referred from (emergency department, directly referred by the GP, referred by another hospital department), stay in the geriatrics department, length of stay, number of medications per prescription at admission and discharge, initiation/discontinuation/adjustment of medication, and whether these changes appeared on the prescription.

Six weeks after DGD, the following data were collected for each patient: drug changes during hospitalization notified on the hospital report (HR), number of medications per prescription, the medication that was changed, type of change (initiation/discontinuation/adjustment), and the motive behind the change.

In order to reflect the usual conditions of care based on existing medical knowledge, all decisions regarding drug and dose changes were left to the discretion of the geriatrician (a senior doctor) and intern (postgraduate medical student) who managed the patient during hospitalization, then to the GP six weeks after DGD [13].

### 2.4. Main Outcomes

The main outcome was the prescription modification six weeks after discharge. Prescription modification is defined as the addition, discontinuation, or dose adjustment of one or more drugs.

### 2.5. Statistical Analyses

Continuous variables, expressed as means ± standard deviations (SD), were compared using nonparametric tests. Categorical variables, expressed as absolute numbers and/or percentages, were compared using the chi2 test or the Fisher’s exact test. Statistical significance was defined as *p* < 0.05.

Prescriptions were compared at three times: before admission to hospital, at DGD and six weeks after DGD.

In order to identify the factors influencing prescription changes at 6 weeks, bivariate and multivariate analyses (by logistic regression) were also performed. After bivariate analysis, variables with a significance level *p* <0.10 were selected and tested together in a multivariate model with the calculation of odds ratios (OR) and 95% confidence intervals (95% CI). The aim was to determine their potential independent effect on the occurrence of drug changes six weeks after DGD.

SAS^®^ 9.4 software (SAS^®^ 9.4, SAS Institute Inc., Cary, NC, USA) was used to conduct all statistical analyses.

## 3. Results

### 3.1. Patients Included

During the study period, 447 patients were discharged from the target departments: 327 (73.2%) from AGU and 120 (26.8%) from GRU.

**Figure 1:**
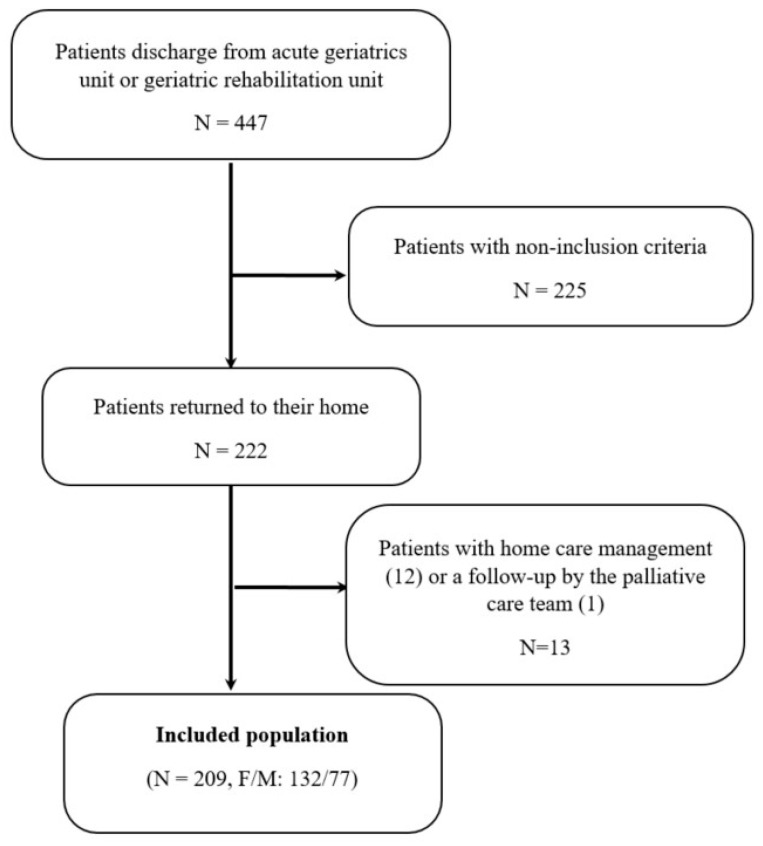
Flowchart of inclusions.

Among the 447 patients, 222 (49.7%) returned to their home. We excluded 13 (2.9%) who had home care management (12 cases) or follow-up with the palliative care team (one case). Finally, 209 patients were included. They were 63.2% female, aged 86.8 ± 6.0 years (66 to 107) (Figure 1).

### 3.2. Characteristics of Patients at Admission

Before hospitalization, 158 of 222 patients (75.6%) lived at home and 51 in a nursing home (24.4%).

The mean number of medications (MNM) per prescription was 7.1 ± 2.9 (0 to 18, median 7).

The most commonly prescribed drugs were paracetamol, ONS, antiplatelet agents, laxatives, benzodiazepines, hypnotics, selective serotonin reuptake inhibitors, beta-blockers, other antidepressants (mianserin, mirtazapine), angiotensin-converting enzyme (ACE) inhibitors, angiotensin II receptor blockers, calcium-channel blockers, thiazide diuretics, amiodarone, proton pump inhibitors, nitrates, oral and inhaled corticosteroids, vitamin K antagonist, direct oral anticoagulants, glinides, biguanides, sulfonylurea drugs, other oral hypoglycemic agents, heparins, cholinesterase inhibitors, mild opioid analgesics, strong opioid analgesics, dopaminergic drugs, other antiparkinson drugs, vitamin D, calcium, bisphosphonates, digitalis, loop diuretics, statins, insulins, allopurinol, antivertigo drugs, thyroid hormones, hydrocortisone, beta-2 mimetics, antispasmodics, antiepileptic medications, antiemetics, centrally acting antihypertensive drugs, antipsychotic drugs, tricyclic antidepressants, antihistamines, nonsteroidal anti-inflammatory drugs, spironolactone, and monoamine oxidase inhibitors.

139 patients (66.5%) were hospitalized in the geriatrics department after admission to the emergency department, 44 (21.0%) were directly referred by their GP, and 26 (12.4%) were referred by other departments.

The main cause of admission to AGU was an infection, while it was a fall for GRU.

The mean length of stay was 28.0 ± 28.7 days (1 to 180) for all patients, 10.0 ± 5.6 (1 to 33) in AGU alone, 56.0 ± 29.0 (22 to 180) for those hospitalized in AGU/GRU, and 39.0 ± 29.1 (14 to 132) in GRU alone.

At discharge, 117 patients (56.0%) returned home and 92 (44.0%) were sent to a nursing home. There were 41 (19.6%) new admissions to a nursing home.

The MNM per prescription at DGD was 7.8 ± 2.6 (1 to 19, median 8). It was significantly higher than the number of medications per prescription at admission (7.8 vs. 7.1, *p* = 0.003).

### 3.3. Patient Characteristics Six Weeks after Hospital Discharge

Six weeks after discharge, 24 patients (11.5%) had been readmitted, 14 (6.7%) died, nine (4.3%) had no contact with their GP, and six (2.9%) were lost to follow up. Thus, 156 patients (74.6%) completed the study.

Six weeks after discharge, the MNM per prescription was 7.72 ± 2.68 (0 to 19, median 8), with no significant difference compared with the numbers recorded at DGD (*p* = 0.529).

### 3.4. Analysis of Prescription Modifications

#### 3.4.1. Prescription Changes during Hospitalization

The prescriptions of 202 patients (96.7%) were changed during their stay. Drug changes were explained in 196 HRs (97.0%). For 160 patients, one or more drugs were stopped, and this interruption was notified on 60 prescriptions (37.5%).

Overall, there were 1217 individual drug modifications during hospitalization (MNM changed per patient: 5.8): 52.8% of these were initiations (MNM initiated per patient: 3.1), 39.3% were interruptions (MNM discontinued per patient: 2.3), and 7.9% were adjustments (MNM with dose adjustment per patient: 0.4).

##### Initiations

ONS (16.0%), laxatives (12.3%), paracetamol (11%), drugs for alimentary tract and metabolism (DATM) (8.1%), and short-acting benzodiazepines (SAB) (6.7%) were the most commonly initiated drugs (Table 1).

Drug initiations were related to several reasons and mainly the treatment of new or old but previously untreated pathology(s), syndrome(s), or symptom(s), as well as changing from one medication that is ineffective, inappropriate, or causing side effects for another.

##### Discontinuations

DATM (7.3%), loop diuretics (7.1%), calcium-channel blockers (4.4%), beta-blockers (4.4%) and SAB (4.0%) were the most commonly discontinued drugs (Table 2).

Drug discontinuations were mainly related to their ineffectiveness, inappropriateness, or side effects.

##### Adjustments

The 96 dose adjustments included beta-blockers (20.8%), loop diuretics (13.5%), ACE inhibitors (13.5%), angiotensin II receptor blockers (8.3%), and proton pump inhibitors (8.3%) (Table 3).

Dose adjustments were made either because of an overdose (for reductions), or suboptimal efficacy (for increases).

#### 3.4.2. Prescription Changes Six Weeks after Hospital Discharge

Six weeks after discharge, 81 patients (51.9%) had no change in their prescriptions. As shown in Table 4, univariate analysis found that six weeks after DGD, there were significantly more drug changes for patients with cognitive impairment (*p* = 0.04) or patients for whom in-hospital modifications were not notified on the HR (*p* = 0.007). Bivariate analysis found that six weeks after DGD, there were significantly more drug changes for patients with cognitive impairment (*p* = 0.037), chronic nephropathy (*p* = 0.019), or patients for whom there were changes in prescription during hospitalization (*p* < 0.001), or in-hospital modifications were not notified on the HR (*p* = 0.005). After multivariate analysis, changes in prescription during hospitalization were the only significant factor for determining the prescription changes six weeks after DGD (*p* < 0.001; OR: 10^4^ (95% CI: 167.99−10^6^).

A total of 169 medications were changed (MNM changed per patient, 1.1), among which 89 (52.7%) were stopped (MNM discontinued per patient: 0.6), 58 were (34.3%) initiated (MNM initiated per patient, 0.4), and 22 (13.0%) were adjusted (MNM with adjusted dose per patient: 0.1).

Therapeutic changes were mainly initiated upon patient request (69 cases, 40.8%). In 44 cases (26.0%), clinical decompensation (i.e., disruption of somatic or psychological health) justified the change in drug regimen. When there had been a change initiated by the GP, they indicated either that there had been a lack of information about outpatient treatment in 30 cases (17.8%), or that they disagreed with the prescription or dosage in 24 cases and or because of side effects in two cases.

##### Initiations

SAB (8.7%), loop diuretics (8.7%), selective serotonin reuptake inhibitors (6.9%), beta-2 mimetics, ACE inhibitors, DATM, and antipsychotic drugs (5.2% each) were the most commonly initiated drugs (Table 5).

The modification in drug regimen was justified by clinical decompensation in 30 of the 58 initiations (51.7%). GPs indicated that they had received insufficient information on outpatient treatment in 22 cases. A new drug was prescribed at patient request in six cases.

##### Discontinuations

ONS (33.7%), SAB (12.4%), laxatives (11.2%), paracetamol (6.7%), DATM (5.6%), other antidepressants (4.5%), calcium, ACE inhibitors, vitamin D (3.4% each), strong opioid analgesics, calcium-channel blockers, proton pump inhibitors, statins (2.2% each), antiplatelets, beta-2 mimetics, beta-blockers, glinides and antipsychotic drugs (1.1% each) were the most commonly discontinued drugs. The discontinuation of ONS was the only change for nine patients.

The patient had requested a discontinuation in 62 of the 89 cases (69.7%). We have no further details on the reasons behind these requests. In 21 cases, the GP considered the prescription inappropriate. In four cases, the GP pointed the lack of information on the outpatient treatment and in two cases the side effects led the GP to interrupt the treatment.

##### Dosage Adjustment

The drug classes that were most commonly adjusted were loop diuretics (31.8%), SAB (13.6%), other antidepressants, ACE inhibitors, antipsychotic drugs (9.1% each), angiotensin II receptor blockers, antiplatelets, antiepileptics, beta-blockers, selective serotonin reuptake inhibitors, and proton pump inhibitors (4.5% each).

Clinical decompensation justified the adjustment in 14 of the 22 cases (63.6%). The GP pointed the lack of information on the outpatient treatment in four cases. In three cases, the GP considered the prescription inappropriate and in one case, the patient requested a dose adjustment.

##### Continuation of In-Hospital Changes

Among the 1217 changes made to prescriptions during hospitalization, 1056 (86.7%) persisted at six weeks including 526 initiations (81.8%), 443 discontinuations (92.7%) and 87 dose adjustments (90.6%).

In eight cases, the GP changed a medication that had not been changed during hospitalization.

## 4. Discussion

To our knowledge, relatively few studies have been published on the subject of changes to drug prescriptions in very elderly patients.

We found a high mortality rate (6.7%) six weeks after hospital discharge. This could be explained by the very advanced age of patients included in the study (mean age 86.8 ± 6 years, max 107 years).

In a study of 1306 patients over 75 years admitted via the emergency department, Dramé et al., found a mortality rate of 10.6% at six weeks, higher than that of our study [14]. However, their population is not fully comparable to ours. Indeed, in our study, 21.0% of patients were directly admitted to a geriatrics department, while in their study, all subjects were admitted to various departments via the emergency department. In addition, our study included only geriatrics departments.

Our study found a MNM per prescription greater than seven, confirming the polypharmacy trend in the elderly. Despite 478 discontinuations, the MNM per prescription was significantly higher at DGD than at admission (7.8 vs. 7.1, *p* = 0.003). This increase proves that therapeutic optimization does not always make it possible to reduce the number of drugs. Moreover, the inclusion of ONS in our study may explain this increase.

Several studies have shown that hospitalization is responsible for a number of changes in the patient’s usual treatment, whether voluntary or not [10,15,16,17]. This result is confirmed by our study. More specifically, the MNM changed per patient was 5.8, and almost all prescriptions were modified (96.7%). In a similar study including 206 subjects with a mean age of 85 years, Rouch et al., found the same change o, MNM per patient (5.5 ± 2.8) during hospitalization [10]. At the admission to the AGU of our university hospital, medication reconciliation is done in order to precisely identify the patient’s usual treatment at home. Thus, we can assume that the changes made on the patient prescription at DGD are not errors or omissions but intentional, relevant changes.

In our study, ONS were the drugs most often initiated at the hospital (16.0%), which confirms that these medications are often prescribed by the hospital care team. We chose to include ONS in our analysis because malnutrition is a major problem in the elderly population [18], and ONS is recommended for severe malnutrition and/or poor dietary intake. ONS were also the drugs most often discontinued at home (33.7%). Nonadherence to ONS must lead us to reconsider how they are prescribed.

We found frequent initiation of SAB during hospitalization (6.7%) even if these drugs have significant side effects in elderly patients. The purpose of this research was not to assess the relevance of the changes, but initiations often replace other treatments which have become inappropriate [19,20]. For example, antipsychotic drugs were stopped in 12 cases, antihistamines in 16 cases, hypnotics in seven cases, and long-acting benzodiazepines in four cases. SAB may be initiated as a result of hospitalization-related stress, and, in this case, must be stopped at discharge. However, interruption is not possible immediately if the patient has become accustomed to the drug. This could explain the 11 discontinuations of SAB six weeks after discharge.

In addition to ONS and SAB, laxatives and paracetamol were the other commonly introduced drugs during hospitalization, which is consistent with the fact that malnutrition [18], pain [21] and constipation [22] are major problems in the elderly. Except for ONS, which was not analyzed, our results are similar to those of a French study that assessed drug modifications among geriatric inpatients [8].

In our population, the rate of unplanned rehospitalizations was relatively high (11.5%). This is not surprising considering the advanced age of the patients included. Morandi et al., found a lower rate of rehospitalizations (4.0%) [23], but their study was performed on relatively younger patients (median 80 years) in a rehabilitation unit, and rehospitalization was studied over 30 days (vs. 45 days in our study). On the contrary, another study performed in persons aged over 75 years found a 30-day rate of unplanned rehospitalizations of 24.6% [24].

Six weeks after discharge, there was no change in prescription for 81 patients (51.9%). This result may seem weak. However, if we focus on the content of the prescriptions, there were only 169 modifications, which corresponds to 1.1 drugs per patient on average. Six weeks after discharge, 86.7% of the prescription changes made during hospitalization were maintained. Another recent study found a similar rate (83%) [10]. These high rates confirms that the drugs prescribed in hospital are continued after discharge in the vast majority of cases. To explain the high rate of GP adherence to drug changes made in hospital, some authors point to the collaboration between geriatricians and hospital pharmacists and the fact that drug regimen modifications were systematically justified in the HR [10]. Again, these observations highlight the major importance of medication reconciliation.

In our study, we found that clinical decompensation (26.0%), miscommunication between the primary sector and the hospital (17.8%), patient request (40.8%), inappropriateness of prescription and occurrence of side effects were the reasons for drug changes six weeks after DGD.

The need to adjust treatment following clinical decompensation is obvious, since clinical decompensation consists of a disturbance in physical or mental health that is most common in frail elderly people. It can be due to the occurrence of an acute factor which may be intrinsic, such as infection or myocardial infarction, or extrinsic, such as grief [25]. Thus, the frailty of the elderly could explain these drug changes in part.

A recent study including geriatric patients found the miscommunication between the primary sector and the hospital to be the main reason for the poor continuity of medication changes by GPs after hospital discharge [11]. Miscommunication between hospital and community health care professionals is common and may adversely affect patient care [26]. The impact is reflected in our study because there were significantly more changes at six weeks when in-hospital modifications were not notified. This kind of miscommunication between hospitals and GPs may also explain why multivariate analysis found more drug changes six weeks after DGD in patients for whom there were changes in prescription during hospitalization. Indeed, if the changes are unjustified, it is more likely that the prescription will be discontinued.

In our study, prescriptions were often changed at the patient’s request (40.8%). Informing and empowering the patient is key to limiting patients’ requests for drug discontinuation and increasing adherence. However, therapeutic adherence is a frequent issue among elderly individuals, and it is probably not sufficiently addressed during hospitalization. Adherence is an important problem in health care because 21% of medical accidents may be attributable to nonadherence [27]. Nonadherence to medication is related to several factors including a higher prevalence of chronic diseases, polypharmacy, and age-related physical and mental capabilities [5]. Disease severity may improve treatment adherence [28] whereas certain comorbidities such as depression, cognitive impairment or high blood pressure increase the risk of nonadherence [29,30,31]. In our study, there were significantly more changes when the patient had cognitive impairment. However, some of these patients lived in a nursing home and did not manage their own medications. Behavioral disorders in patients with cognitive disorders may also explain some therapeutic changes. Finally, we found that nonadherence was pronounced for ONS, which may be explained by asymptomatic nature of malnutrition.

Drug changes for a prescription that was deemed inappropriate can be explained by the fact that GPs are made aware of the rules for prescribing and apply these rules carefully [6,7]. In some cases and for a given patient, a drug may be appropriate at one time (during hospitalization for example) and then become inappropriate later (at home for example).

The occurrence of side effects as factor of drug changes is also explained by GPs applying the rules of good drug prescription. Several factors can cause side effects, including drug overdose, acute kidney or liver failure, diet change, and drug-drug interactions because of polypharmacy, the last being very common in older patients [32].

Our study has some limits. In order to assess changes in prescription, we established a list of the most prescribed drugs. Potassium, folate, and vitamin B_12_ were not included in that list though these drugs were often prescribed during our study. These were the main drugs found in the DATM class, which was one of the first five drug classes found in discontinuations and initiations during hospitalization and at six weeks. Information regarding prescriptions was collected over the phone six weeks after discharge, which could lead to a loss of information. However, we chose this method in order to guarantee a high rate of follow-up at six weeks.

Our study offers promising prospects. For instance, a high proportion of patients asked to discontinue certain drugs, so future studies could focus on the reasons behind these types of requests. Understanding the reasons for patient-initiated discontinuations will make it easier to suitably educate patients by explaining, for example, the importance of the drug (in order to increase adherence) or what to do in the event of side effects.

Communication between hospital physicians and GPs, which is essential for optimal continuity of patient care, should be regularly evaluated. Indeed, we found that the insufficiency or lack of information regarding outpatient treatment in the HR was one main reason for prescription changes by the GP six week after hospital discharge.

Finally, it is important to underscore the contribution of pharmacists to therapeutic adherence and the fight against self-medication in the elderly, thanks to the many opportunities they have to advise, alert and monitor patient behavior. Better recognition of this important role would strengthen physician-pharmacist collaboration and communication, giving pharmacists a more prominent position in patient management.

## 5. Conclusions

Our work confirms that nearly every patient hospitalized in a geriatrics department was confronted with at least one change in their treatment regime, and shows that the changes initiated in hospital were generally maintained after the patient returned home. The frailty of elderly patients may explain part of the home drug changes. It is necessary to improve patient adherence and hospital-GP communication in order to promote continuity of care and to optimize patient supervision after hospital discharge.

Optimizing all aspects of patient care—including adherence to treatment, patient education about side effects, and knowledge of the risks of self-medication, and providing better continuity of care require close collaboration between the hospital physician and the GP, but also the involvement of other health professionals such as pharmacists and nurses. In addition, family caregivers should be included in the medical management of the patient, with his/her consent. High-quality communication between the various health professionals and with family caregivers is therefore essential.

## Figures and Tables

**Table 1:** Drug initiations during the hospitalization.

Drug Classes	Number of Initiations	%
Oral nutritional supplements	103	16.0
Laxatives	79	12.3
Paracetamol	71	11
Alimentary tract and metabolism	52	8.1
Short acting benzodiazepines	43	6.7
Vitamin D	31	4.8
Other antidepressants	23	3.6
Selective serotonin reuptake inhibitors	21	3.3
Proton pump inhibitors	17	2.6
Calcium	16	2.5
Angiotensin-converting enzyme inhibitors	16	2.5
Antiplatelet medications	14	2.2
Insulin	14	2.2
Strong opioid analgesics	12	1.9
Calcium-channel blockers	12	1.9
Antipsychotic drugs	9	1.4
Vitamin K antagonist	8	1.2
Cholinesterase inhibitors	8	1.2
Angiotensin II receptor blockers	7	1.1
Direct oral anticoagulants	7	1.1
Hypnotics	7	1.1
Beta-blockers	7	1.1
Loop diuretics	6	0.9
Beta-2 mimetics	6	0.9
Antiepileptic medications	5	0.8
Glinides	4	0.6
Heparins	4	0.6
Statins	4	0.6
Genito-urinary system and sex hormones	4	0.6
Nervous system	4	0.6
Corticosteroids	3	0.5
Digitalis	3	0.5
Antiemetics	2	0.3
Other oral antidiabetic drugs	2	0.3
Dopamine	2	0.3
Cardiovascular system	2	0.3
Musculoskeletal system	2	0.3
Mild opioid analgesics	1	0.2
Antihistamines	1	0.2
Centrally acting antihypertensive drugs	1	0.2
Antispasmodics	1	0.2
Antivertigo drugs	1	0.2
Long acting benzodiazepines	1	0.2
Biguanides	1	0.2
Inhaled corticosteroids	1	0.2
Nitrates	1	0.2
Systemic hormonal preparation (excluding sex hormones and insulins)	1	0.2
Thyroid hormones	1	0.2
Blood and blood-forming organs	1	0.2
Respiratory system	1	0.2

**Table 2:** Drug discontinuations during the hospitalization.

Drug Classes	Number of Discontinuations	%
Alimentary tract and metabolism	35	7.3
Loop diuretics	34	7.1
Calcium-channel blockers	21	4.4
Beta-blockers	21	4.4
Short acting benzodiazepines	19	4.0
Antiplatelet medications	18	3.8
Mild opioid analgesics	17	3.6
Antihistamines	16	3.3
Statins	16	3.3
Vitamin K antagonist	15	3.1
Angiotensin II receptor blockers	14	2.9
Selective serotonin reuptake inhibitors	14	2.9
Angiotensin-conversion enzyme inhibitors	12	2.5
Proton pump inhibitors	12	2.5
Antipsychotic drugs	12	2.5
Vitamin D	12	2.5
Paracetamol	11	2.3
Genito-urinary system and sex hormones	11	2.3
Biguanides	9	1.9
Amiodarone	9	1.9
Hypoglycemic sulfamides	8	1.7
Allopurinol	7	1.5
Antivertigo drugs	7	1.5
Beta-2 mimetics	7	1.5
Digitalis	7	1.5
Thiazide diuretics	7	1.5
Hypnotics	7	1.5
Calcium	6	1.3
Laxatives	6	1.3
Strong opioid analgesics	5	1.0
Centrally acting antihypertensive drugs	5	1.0
Other oral antidiabetic drugs	5	1.0
Bisphosphonates	5	1.0
Insulins	5	1.0
Cardiovascular system	5	1.0
Aldosterone receptor antagonists	4	0.8
Cholinesterase inhibitors	4	0.8
Antispasmodics	4	0.8
Other antiparkinson drugs	4	0.8
Long acting benzodiazepines	4	0.8
Oral nutritional supplements	4	0.8
Antiepileptic medications	3	0.6
Corticosteroids	3	0.6
Imipramine	3	0.6
Musculoskeletal system	3	0.6
Nervous system	3	0.6
Antiemetics	3	0.6
Non-steroidal anti-inflammatory drugs	2	0.4
Direct oral anticoagulants	2	0.4
Other antidepressants (mianserin, mirtazapine, …)	2	0.4
Nitrates	2	0.4
Glinides	2	0.4
Heparins	2	0.4
Various	1	0.2
Thyroid hormones	1	0.2
Monoamine oxidase inhibitors	1	0.2
Respiratory system	1	0.2

**Table 3:** Dosage modifications of drugs during the hospitalization.

Drug Classes	Number of Dose Adjustments	%
Beta-blockers	20	20.8
Loop diuretics	13	13.5
Angiotensin-converting enzyme inhibitors	13	13.5
Angiotensin II receptor blockers	8	8.3
Proton pump inhibitors	8	8.3
Short acting benzodiazepines	7	7.3
Thyroid hormones	5	5.2
Dopamine	3	3.1
Selective serotonin reuptake inhibitors	3	3.1
Strong opioid analgesics	2	2.1
Antiplatelet medications	2	2.1
Other antidepressants (mianserin, mirtazapine, …)	2	2.1
Calcium-channel blockers	2	2.1
Statins	2	2.1
Cholinesterase inhibitors	1	1.0
Antiepileptic medications	1	1.0
Corticosteroids	1	1.0
Glinides	1	1.0
Insulins	1	1.0
Antipsychotic drugs	1	1.0

**Table 4:** Factors influencing prescription changes at 6 weeks, after univariate, bivariate and multivariate (logistic regression) analyses.

Analyzed Factors		*p*	p *	p **	OR (95% CI)
Age		0.18	0.426		
Gender		0.40	0.502		
Residential status before hospitalization		0.35	0.264		
Medical history	Cognitive impairment	0.04	0.037	0.718	1.19 (0.16–9.02)
	Depression	0.17	0.089	0.350	2.94 (0.26–33.79)
	Hypertension	0.68	0.425		
	Heart failure	0.18	0.404		
	Atrial fibrillation	0.32	0.246		
	Diabetes	0.60	0.485		
	Thyroid dysfunction	0.58	0.835		
	Chronic nephropathy	0.08	0.019	0.565	0.62 (0.06–6.72)
Number of drugs at admission		0.86	0.638		
Patient provenance		0.58	0.408		
Type of stay		0.89	0.632		
Unit		0.07	0.049		
Length of stay		0.75	0.732		
Residential status after hospitalization		0.08	0.148		
Number of drugs/prescription at discharge		0.30	0.419		
Changes in prescription during hospitalization		0.21	<0.001	<0.001	10^4^ (167.99–10^6^)
Changes reported on discharge prescription		0.17	0.159		
In-hospital modification(s) notified on hospital record		0.007	0.005	0.571	10^−4^ (10^−5^–10^5^)

*p*, *p*-value corresponding to univariate analysis; *p* *: p-value observed after bivariate analysis; *p* **, *p*-value after multivariate analysis using logistic regression; OR, odds ratio; CI, confidence interval.

**Table 5:** Drug initiations 6 weeks after hospital discharge.

Drug Classes	Number of Initiations	%
Short acting benzodiazepines	5	8.6
Loop diuretics	5	8.6
Selective serotonin reuptake inhibitors	4	6.9
Alimentary tract and metabolism drugs	4	6.9
Beta-2 mimetics	3	5.2
Angiotensin-converting enzyme-inhibitors	3	5.2
Antipsychotic drugs	3	5.2
Mild opioid analgesics	2	3.4
Antiplatelet medications	2	3.4
Thiazide diuretics	2	3.4
Proton pump inhibitors	2	3.4
Vitamin D	2	3.4
Angiotensin II receptor blockers	1	1.7
Vitamin K antagonist	1	1.7
Allopurinol	1	1.7
Paracetamol	1	1.7
Strong opioid analgesics	1	1.7
Antihistamines	1	1.7
Antivertigo drugs	1	1.7
Antiepileptic medications	1	1.7
Other antidepressants (mianserin, mirtazapine, …)	1	1.7
Long acting benzodiazepines	1	1.7
Beta-blockers	1	1.7
Oral nutritional supplements	1	1.7
Dopamine	1	1.7
Nitrates	1	1.7
Hypnotics	1	1.7
Calcium-channel blockers	1	1.7
Insulin	1	1.7
Laxatives	1	1.7
Hypoglycemic sulfamides	1	1.7
Genito-urinary system and sex hormones	1	1.7
Musculoskeletal system drugs	1	1.7
